# Dynamic Bayesian network structure learning based on an improved bacterial foraging optimization algorithm

**DOI:** 10.1038/s41598-024-58806-0

**Published:** 2024-04-09

**Authors:** Guanglei Meng, Zelin Cong, Tingting Li, Chenguang Wang, Mingzhe Zhou, Biao Wang

**Affiliations:** 1 School of Automation, Shenyang Aerospace University, Shenyang, 110136 China; 2 Aviation Science and Technology Key Laboratory of Air Combat System Technology, Shenyang, 110136 China

**Keywords:** Dynamic Bayesian networks, Structural learning, Swarm intelligence optimization algorithm, Bacterial foraging optimization algorithm, Engineering, Mathematics and computing

## Abstract

With the rapid development of artificial intelligence and data science, Dynamic Bayesian Network (DBN), as an effective probabilistic graphical model, has been widely used in many engineering fields. And swarm intelligence algorithm is an optimization algorithm based on natural selection with the characteristics of distributed, self-organization and robustness. By applying the high-performance swarm intelligence algorithm to DBN structure learning, we can fully utilize the algorithm's global search capability to effectively process time-based data, improve the efficiency of network generation and the accuracy of network structure. This study proposes an improved bacterial foraging optimization algorithm (IBFO-A) to solve the problems of random step size, limited group communication, and the inability to maintain a balance between global and local searching. The IBFO-A algorithm framework comprises four layers. First, population initialization is achieved using a logistics-sine chaotic mapping strategy as the basis for global optimization. Second, the activity strategy of a colony foraging trend is constructed by combining the exploration phase of the Osprey optimization algorithm. Subsequently, the strategy of bacterial colony propagation is improved using a "genetic" approach and the Multi-point crossover operator. Finally, the elimination-dispersal activity strategy is employed to escape the local optimal solution. To solve the problem of complex DBN learning structures due to the introduction of time information, a DBN structure learning method called IBFO-D, which is based on the IBFO-A algorithm framework, is proposed. IBFO-D determines the edge direction of the structure by combining the dynamic K2 scoring function, the designed V-structure orientation rule, and the trend activity strategy. Then, according to the improved reproductive activity strategy, the concept of "survival of the fittest" is applied to the network candidate solution while maintaining species diversity. Finally, the global optimal network structure with the highest score is obtained based on the elimination-dispersal activity strategy. Multiple tests and comparison experiments were conducted on 10 sets of benchmark test functions, two non-temporal and temporal data types, and six data samples of two benchmark 2T-BN networks to evaluate and analyze the optimization performance and structure learning ability of the proposed algorithm under various data types. The experimental results demonstrated that IBFO-A exhibits good convergence, stability, and accuracy, whereas IBFO-D is an effective approach for learning DBN structures from data and has practical value for engineering applications.

## Introduction

Bayesian networks (BNs) combine probability theory with graph theory and exhibit strong interpretability and high learning efficiency. The logical relationships inherent in data can be effectively investigated, offering a promising technical approach for establishing causal models for complex problems^1^. Dynamic Bayesian networks (DBNs) are powerful algorithmic tools that integrate the structure of static BNs with time-related information and are employed for dynamic uncertainty inference and temporal data analysis. DBNs have applications in various fields, including artificial intelligence, machine learning, and automatic control^2^. Furthermore, DBNs have a broad range of engineering applications, such as managing transcriptional regulatory relationships between cancer genes^3^, identifying connectivity issues between human brain regions through high-order DBNs using functional magnetic resonance imaging time series data^4^, and analyzing the vascularization in the formation process of engineered tissues, aiming to enhance the accuracy of predicting future time steps and ensuring an acceptable uncertainty in forecasting the future progress of the organization^5^. Integrating structural prediction methods, such as mutual information and maximum information coefficient into the DBN model enhances the efficiency and scale of gene regulatory network reconstruction^6^.

However, the introduction of time information into the DBN network increases search space complexity, reduces structure learning accuracy, and impedes the direct application of the static BN learning method. Early learning approaches involved constructing DBN network structures by experts based on their prior experience^7,8^. Conversely, for large datasets with numerous parameters and high complexity, relying only on expert experience poses challenges in establishing causal relationships based on temporal information and ensuring structural accuracy. To solve these problems, Leray et al.^9^ proposed a classical DBN structure learning algorithm inspired by the dynamic max–min hill climb (DMMHC) local search algorithm. This algorithm allows for the quick identification of constraints based on temporal information in the 2T-BN model. Trabelsi et al.^10^ introduced the heuristic greedy search (GS) algorithm^11^, which was extended to 2T-BN networks to perform local optimal selection through a generative neighborhood algorithm as a way to expect to find the globally optimal DBN structure.

However, these classical DBN structure learning methods still have problems such as low efficiency of generating networks and lack of escaping local optimum mechanism. Therefore, how to further optimise the DBN structure learning methods to improve their search efficiency and search accuracy is the current research hotspot for scholars. In recent years, researchers have shown significant interest in nature-inspired metaheuristic algorithms based on swarm intelligence (SI) for optimization. Notable optimization algorithms include genetic algorithm (GA)^12^, ant colony optimization^13^, particle swarm optimization (PSO)^14,15^, grey wolf optimization (GWO)^16^, artificial bee colony^17^, bat optimization^18^, whale optimization^19^, and firefly optimization^20^. Scholars have also improved the aspects of population initialization and individual optimization iterative updating strategies of these optimization algorithms on the original basis by introducing a series of effective methods such as Lévy flights^21^, opposition-based learining^22^, adaptive strategies, and hybrid algorithms, aiming to improve their optimization performance, as well as the accuracy and stability of the optimization results. Wu et al.^23^ proposed an improved ant colony optimization algorithm (ICMPACO) by introducing multiple swarm strategies, co-evolutionary mechanism, pheromone updating strategy and pheromone diffusion mechanism, which improved the optimization ability and stability of the algorithm. And it was applied to Traveling Salesmen Problem to obtain better allocation results. Gao et al.^24^ proposed an improved variable weight gray wolf optimization algorithm VW-GWO, which improves the probability of the algorithm escaping from the local optimum by introducing control parameters and decreasing control equations. Liu et al.^25^ proposed an adaptive weighted particle swarm optimization algorithm (AWPSO) based on sigmoid function, which updated the acceleration coefficients using the sigmoid activation function of the neural network and considered both the particles to the global optimal position through an adaptive weighting strategy, thus improving the optimization efficiency and convergence characteristics of the algorithm. Zhang et al.^26^ proposed a Chaotic Bacterial Foraging Optimization (ChaoticBFO) algorithm to achieve a reasonable balance between exploration and exploitation by introducing a chaotic initialization strategy and a chaotic local search with a "contraction" strategy in the convergence step. Mou et al.^27^ proposed an adaptive non-dominated sorting genetic algorithm III (ANSGAIII), the algorithm enhances the objective function by considering non-linear relationships, equality constraints, actuator rate and position constraints. The algorithm solves the problem of autonomous berthing and dynamic positioning of over-actuated ships. Giri et al.^28^ proposed an adaptive neighbourhood for locally and globally tuned biogeography based optimization algorithm (ANLGBBO) which inherits features of the nearest neighbour of the local best individual to be migrated along with a global best individual of the pool. And explore large Spaces by identifying areas with high-quality solutions. In addition, literature research shows that advanced optimization algorithms and their variants have played an important role in practical engineering applications in many intelligent fields, such as: Data mining engineering^29,30^, automatic driving^31,32^, intelligent robots^33^, network topology architecture^34,35^, military intelligent equipment^36,37^ and other fields.

The use of metaheuristic search mechanisms to investigate the solution space is a common characteristic among SI-based algorithms. During each computation iteration, these algorithms update the state of the local models and generate new populations to search for local optima. The quality of the solutions continuously improves as the number of iterations increases, ultimately approaching the optimal model. Based on this, optimizing the DBN network structure using a high-performance swarm intelligence optimization algorithm is a novel and effective research method. Li et al.^38^ introduced a binary PSO algorithm based on mutual information, which effectively prunes the search space. The algorithm accelerates the convergence speed of deep belief network structure learning by updating the particles using a probability threshold. Heng et al.^39^ proposed a fitness function and redefined the encoding format of the particle swarm algorithm for DBN structure learning from incomplete datasets. Santos et al.^40^ redefined the positions and velocities in the particle swarm algorithm to learn the high-order dynamic Bayesian network structure from large-scale and multivariate time series data. Jiang et al.^41^ proposed an adaptive learning algorithm for DBN structure learning using a two-step strategy and adaptive crossover and mutation rates within a GA framework. Quesada et al.^42^ proposed an order-invariant, Markov order-independent high-order particle encoding approach that exhibits scalability in high-order networks. Deng et al.^43^ proposed an improved binary bat algorithm for learning the transitional network structure of a DBN and constructed a fitness function to quantitatively assess the node order in the network.

Although the aforementioned SI-based DBN structure learning approaches have achieved a certain level of effectiveness, there is still substantial potential for improvement in terms of optimization efficiency and accuracy. Passino et al.^44^ proposed a bacterial foraging optimization (BFO) algorithm that stimulates the foraging behavior of Escherichia coli bacteria in the human body. BFO is a global stochastic search algorithm, The simulation of the bacterial population comprises four steps: chemotaxis, grouping, reproduction, and elimination-dispersal. It has the characteristics of not requiring the gradient information of the optimization object during the optimization process, low complexity and fast convergence, which can be applied to reduce the number of iterative convergence times for finding the candidate network, jumping out of the local optimum, and searching for the highest scoring globally optimal DBN network structure. However, the original BFO also has certain defects, such as random steps of chemotactic activity, poor information exchangeability of clustering mechanism, and inability to maintain a balance between global and local search.

In summary, to enhance the BFO optimization performance, we propose a new hybrid algorithm, called the improved bacterial foraging optimization algorithm (IBFO-A), which aims to improve optimization iteration speed and accuracy while maintaining the low time complexity and fast convergence performance of BFO and balancing the global and local exploration and development capabilities. Then, within the framework of the IBFO-A algorithm, combined with a dynamic K2 scoring function and customized learning strategy, an IBFO-D method for DBN structure learning is designed to improve its ability to optimize the learning network structure from the data.

The main contributions of this study can be summarized as follows:

1. To improve the population quality, the population is initialized using a logistics-sine chaotic mapping strategy. During the development and exploration phase of hybrid osprey optimization algorithm (OOA), the chemotactic activity of bacteria was reconstructed, improving the ability of individual bacteria to recognize and move toward the optimal target fitness value.
2. Based on the replication idea of GA and the Multi-point crossover operator, the reproduction steps were reconstructed. This involves crossing the poor individual $X_{{{\text{worst}}}}^{{}}$ and fusing the better individual $X_{{{\text{best}}}}^{{}}$, thereby improving performance, increasing the species diversity of the flora, and escaping the local optimal solution based on the elimination-dispersal operator.
3. A dynamic K2 scoring function and V structure orientation rule are established. Combined with the IBFOA framework, the cumulative health score is saved during the breeding stage to reduce the number of iterative convergence in searching for candidate networks, and it is used in the elimination-dispersal stage to find the globally optimal DBN network structure with the highest score.
4. The effectiveness of the proposed algorithms (IBFO-A and IBFO-D) is evaluated and comparisons are made with other mature algorithms through experimental tests on the benchmark data set. Statistical analyses of the experimental results are conducted as follows:
5. Firstly, the IBFO-A algorithm is compared with seven other optimization algorithms using 10 sets of different types of CEC2005 benchmark functions (unimodal, multimodal, and hybrid). These include three original algorithms, two classical algorithms, and two recent advanced algorithms. Additionally, sensitivity analysis experiments were conducted for the three parameters of IBFO-A. The experimental results indicate that IBFO-A algorithm runs stably, ASR ranks first and has a certain competitiveness. Subsequently, we conducted comparative experiments on 12 optimization algorithms, including IBFO-A, using the CEC2019 benchmarking functions and two real-world engineering optimization problems. Some novel as well as improved optimization algorithms are included. The experimental results show that the IBFO-A algorithm exhibits good optimization performance, indicating its potential in real engineering applications.
6. Secondly, the $B_{0}$ and $B_{ \to }$ network structure learning capabilities of IBFO-D in non-temporal and temporal data samples are investigated, revealing that the generated network structure can converge stably within a high fitness value.
7. Finally, IBFO-D is compared with two other structure learning algorithms using six 2T-BN temporal network data samples. The experimental results show that IBFO-D is an effective method for optimizing DBN structure learning from the data.

The remaining sections of this study are structured as follows. Preliminaries offers a review of the relevant concepts and scoring metrics of BN, along with an introduction to the basics of DBN. In Methodology, the principles of IBFO-A and IBFO-D algorithms and the design of dynamic scoring function are described in detail. Experimental section presents the results and analysis of the simulation experiments. Finally, Conclusion provides the conclusion and outlines plans for future research.

## Preliminaries

### Static Bayesian network

The Bayesian network $N$ is represented as a binary tuple $N = (G,\Theta )$ comprising structure $G$ and network parameters $\Theta$. In graph theory, the independent relationships among a set of variables can be represented using a directed acyclic graph (DAG). Here, $G = (X,E)$ represents a specific instance or representation of such a graph; where $X$ is a nonempty set of all nodes in the graph. $X = \left\{ {X_{1} ,X_{2} , \ldots ,X_{i} , \ldots ,X_{n} } \right\}$, $X_{i}$ can be either an observed variable or a latent variable; $E$ is the set of directed line segments between different variables in the DAG, and $X_{j} \to X_{i}$ d represents the direct dependencies between nodes^45^.

1 $$ E = \left\{ {X_{j} \to X_{i} |X_{j} \in pa(X_{i} ),i = 1, \ldots ,n} \right\} $$

where $pa(X_{i} )$ is the "causes" of the node $X_{i}$, also called the set of parent nodes. Given the parent node set $pa(X_{i} )$, $X_{i}$ is independent of its non-descendant node set $nd(X_{i} )$ based on Markov independence. Thus, the joint probability of several nodes $X_{i}$ that follow the Markov rule can be expressed as follows:

2 $$ P(X_{1} , \cdots ,X_{n} ) = \prod\limits_{i = 1}^{n} {P(X_{i} |pa(X_{i} ))} $$

The conditional probability table of each node $X_{i}$ given its known parent node set $pa(X_{i} )$ is represented by the network parameter $\Theta = \{ \Theta_{1} ,\Theta_{2} , \ldots ,\Theta_{n} \}$. It is possible to calculate the joint probability distribution of the node $X_{i}$ when the network structure $G$ and the network parameters $\Theta$ of a Bayesian network are known. Compared with other approaches for calculating joint probabilities, the efficiency of Bayesian network algorithms is significantly higher because of the conditional independence among nodes.

### Scoring function

The search and score-based BN learning approach mainly comprises two parts: model selection and model optimization. Its core idea involves considering all possible structures as the domain, selecting a scoring function that assesses the quality of specific structures, and treating the process of identifying the best structure as an optimization problem of searching for the optimal value of the scoring function within the domain.

Prior knowledge about structure $G$ is summarized as a probability distribution $P(G)$, referred to as the structure prior distribution for a Bayesian network $N = (G,\Theta )$. Similarly, prior knowledge about parameters $\Theta$ is summarized as another probability distribution $P(\Theta |G)$ referred to as the parameter prior distribution for a given structure $G$. In this manner, the prior distribution of $N$ can be expressed as follows:

3 $$ P(G,\Theta ) = P(G)P(\Theta |G) $$

The posterior probability distribution $P(G|D)$ is calculated when given an observed dataset $D = \{ D_{1} ,D_{2} , \ldots ,D_{N} \}$. Only the structural models $G^{*}$ corresponding to the maximum posterior probability distribution in the search space are considered.

4 $$ G^{*} = \mathop {\arg \max }\limits_{G} P(G|D) $$

And

5 $$ P(G|D) = \frac{P(D|G)P(G)}{{P(D)}} $$

Selecting the structure with the maximum posterior probability is equivalent to selecting the structure that maximizes the following function since $P(D)$ does not depend on $G$:

6 $$ \mathop {\arg \min }\limits_{G} \log P(G|D) = \mathop {\arg \min }\limits_{G} \log P(D|G) + \log P(G) + C $$

Based on penalized maximum likelihood or marginal likelihood, various scoring metrics, including Bayesian Dirichlet, Bayesian Dirichlet equivalent, K2, minimum description length, Bayesian information criterion, and mutual information test, have been proposed to assess the fitness of networks during the search process.

The most classic K2 scoring function formula is expressed as follows:

7 $$ P(G,D) = P(G)\prod\limits_{i = 1}^{n} {\prod\limits_{j = 1}^{{q_{i} }} {\frac{{(r_{i} - 1)!}}{{(N_{ij} + r_{i} - 1)!}}} } \prod\limits_{k = 1}^{{r_{i} }} {N_{ijk} !} $$

where $n$ denotes the number of variables in the sample, $q_{i}$ denotes the number of parent nodes for $X_{i}$, $r_{i}$ denotes the number of possible values for $X_{i}$, $N_{ijk}$ denotes the number of samples, and $N_{ij}$ denotes the total number of samples.

### Dynamic Bayesian networks

DBN is a graphical model structure that illustrates the conditional independence relationships between random variables and their temporal evolution patterns^46^. Its unique transition network can reflect the state changes of the system under different environmental factors in various time slices, showing the complex interactions and dependencies among variables in the system and offering a closer approximation to the real situations of dynamic multidimensional data.

However, representing $X_{1} ,X_{2} , \ldots ,X_{n}$ stochastic processes using DBN requires deriving a probability distribution over the random variable a, which can be highly complex. Thus, it is crucial to make appropriate assumptions about DBN and design a reasonable and efficient optimization algorithm for structure learning to study and model complex systems (see Methodology). These assumptions can be summarized as follows:

1. The marginal directionality rule describes the dependency relationships between nodes in a finite time slice *t*, and the changes in conditional probabilities tend to converge to consistent stability across all processes.
2. Given the random variables at time step *t*, the random variables at time step $t + 1$ are conditionally independent of the remaining random variables;$X_{t + 1} \underline{||} \left( {X_{t - 1} ,X_{0} } \right)|X_{t}$. In other words, the Markov chain property is satisfied to $P(X_{t + 1} |X_{0} ,X_{1} , \ldots ,X_{t} ) = P(X_{t + 1} |X_{t} )$ by the entire dynamic discrete-time probabilistic process^47^.
3. Across all adjacent time steps, the network topology remains invariant and the transitional network, along with its corresponding conditional probability dependencies, remains the same. In other words, $P(X_{t + 1} |X_{t} )$ is independent of time t.

The DBN constructed on the time trajectory of the random process comprises two components based on the aforementioned conditions: $(B_{0} ,B_{ \to } )$.

1. The initial network $B_{0}$, defined on the initial state $X_{0}$, and the joint probability distribution $P(X_{0} )$ obtained from it form the most initial graphical structure of the Bayesian network (BN) from which the prior probabilities of any node can be derived.
2. The graphical structure of the BN composed of more than two time steps is represented by the transitional network $B_{ \to }$, defined by variables $X_{0}$ and $X_{1}$, with transitional probabilities $P(X_{t + 1} |X_{t} )$.

**Figure 1:**
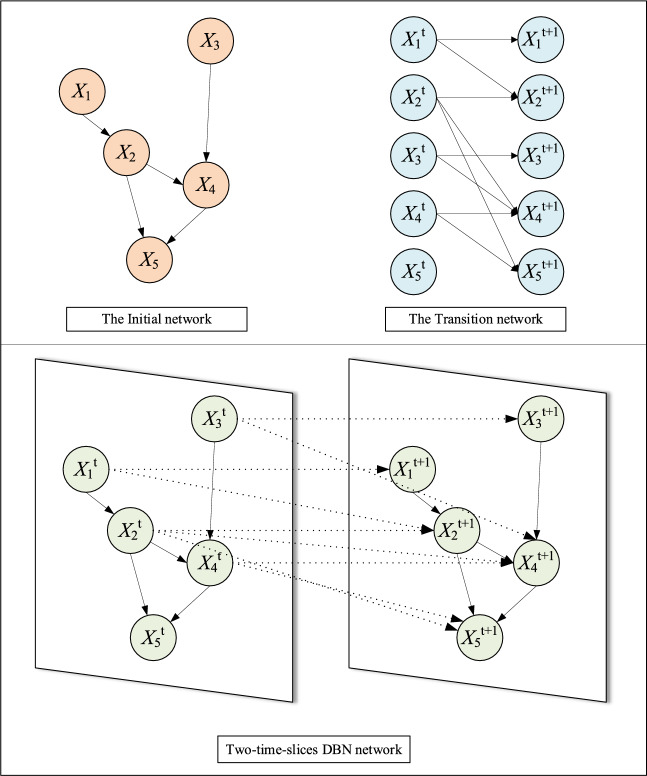
Dynamic Bayesian network model diagram.

In other words, the entire DBN corresponds to $\left\{ {0,1,2, \ldots ,T} \right\}$ finite period, a and unfolds the probabilistic graphical model onto the topology of the random variable $X_{0} ,X_{1} , \ldots ,X_{T}$. The parent nodes of $X_{0}$ are those in the initial network $B_{0}$ at time 0. At time $t + 1$, the parent nodes of $X_{t + 1}$ are those in the transitional network $B_{ \to }$ that are relevant in both time steps $t$ and $t + 1$. A set of initial networks $B_{0}$, a transitional network $B_{ \to }$, and a simple DBN model structure with two time slices are illustrated in Fig. 1.

To summarize, given a DBN model, the joint probability distribution on $X_{0} ,X_{1} , \ldots ,X_{T}$ is defined as follows:

8 $$ P(X_{0} ,X_{1} , \ldots ,X_{T} ) = P_{{B_{0} }} (X_{0} )\prod\limits_{t = 0}^{T} {P_{{B_{ \to } }} } (X_{t + 1} |X_{t} ) $$

**Figure 2:**
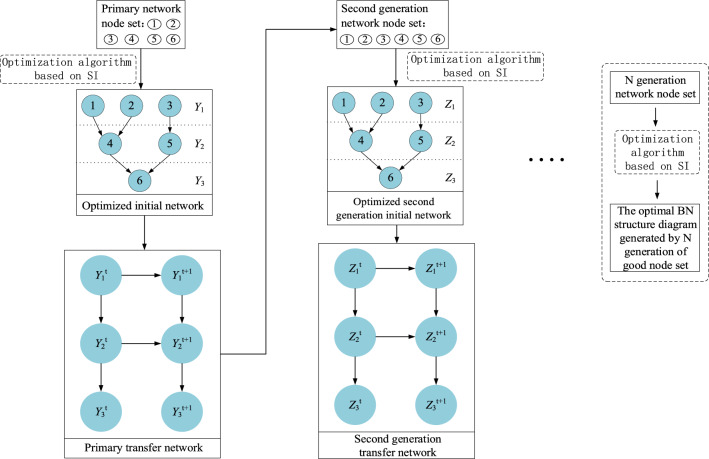
DBN intelligent optimization model diagram.

To solve the actual optimization decision problem, the SI-based structure learning method of DBN extends the static optimization model, starting with the static initial network $B_{0}$. This process involves the construction of a basic graph model for dynamic intelligent optimization using time slice information. Figure 2 shows the specific algorithmic process. A round of the BN network node set can be generated through the transitional network $t + 1$ when the environmental factors change in round $B_{ \to }$. A new population is generated along with the actual optimization problem based on the BN nodes in the round $t + 1$, which is then evolved and optimized to produce a set of excellent solutions and an optimal BN structure graph that matches the current environment, serving as the most suitable reasoning tool for the current problem. Subsequently, the node set to be optimized in the round $t + 2$ is generated by the DBN, and this process continues. As environmental factors change, inference and optimization are conducted to effectively address various emergencies and enhance the mitigation of the effect of uncertain factors on the findings.

## Methodology

### IBFO-A optimization algorithm

#### Population initialization

For swarm intelligent optimization algorithms, the selection of individual initial positions often affects the algorithm's iterative convergence performance. The original BFO algorithm uses random initial locations, which results in the dispersion of most bacteria generated at the initial moment being far away and even not meeting the boundary constraints. Chaotic mapping^48^ is an effective method to improve the population initialization of the optimization algorithm. In this study, a logistics-sine mixed method proposed by Demir et al.^49^ is used to integrate the two most universal methods of chaotic mapping: Logistic mapping and Sine mapping evenly distribute the population in the mapping space, significantly improving the species diversity and search efficiency of the population in the following ways:

The upper bound of the feasible domain of each dimension of the objective function $ub = [ub_{1} ,ub_{2} , \ldots ,ub_{d} ]$ and the lower bound $lb = [lb_{1} ,lb_{2} , \ldots ,lb_{d} ]$. The location matrix modeling of bacterial individuals in the search space is as follows:

9 $$ X = \left[ {\begin{array}{*{20}c} {x_{1,1} } & \cdots & {x_{1,j} } & \cdots & {x_{1,m} } \\ \vdots & \ddots & \vdots & {\mathinner{\mkern2mu\raise1pt\hbox{.}\mkern2mu \raise4pt\hbox{.}\mkern2mu\raise7pt\hbox{.}\mkern1mu}} & \vdots \\ {x_{i,1} } & \cdots & {x_{i,j} } & \cdots & {x_{i,m} } \\ \vdots & {\mathinner{\mkern2mu\raise1pt\hbox{.}\mkern2mu \raise4pt\hbox{.}\mkern2mu\raise7pt\hbox{.}\mkern1mu}} & \vdots & \ddots & \vdots \\ {x_{n,1} } & \cdots & {x_{n,j} } & \cdots & {x_{n,m} } \\ \end{array} } \right]_{ \, n \times m} $$

Use the logistics-sine method to initialize the bacterial individual location:

10 $$ a_{i + 1} = ra_{i} \left( {1 - a_{i} } \right) $$

11 $$ b_{i + 1} = \frac{{\left( {4 - r} \right)\sin \left( {\pi b_{i} } \right)}}{4} $$

12 $$ x_{i + 1} = \left( {a_{i + 1} + b_{i + 1} } \right)\left( {\bmod 1} \right) $$

where $a_{i} \in (0,1)$ and $b_{i} \in (0,1)$ are randomly generated series, $a_{i + 1}$ represents the logistic chaotic mapping, $b_{i + 1}$ represents the sine chaotic mapping, $r$ represents the chaos coefficient, and $x_{i + 1}$ is the bacterial chaotic mapping value determined by logistics-sine.

Finally, the chaotic sequence is mapped to the solution space:

13 $$ X_{i} = lb + x_{i + 1} (ub - lb) $$

#### Chemotactic activity

Chemotactic activity plays a crucial role in the IBFO-A's algorithm, in which bacteria first tentatively choose the direction for a "flip" motion, and then swim to a nutrient-rich area through a "swim" motion. In the original BFO algorithm, the trend activity is randomly given the i th bacterial movement step $C(i)$ and receives the attraction signal from other individuals in the population to swim to the center of the population, and the attraction between bacteria is represented by ${\text{J}}_{{{\text{cc}}}}^{{\text{i}}} \left( {\theta ,\theta^{{\text{i}}} ({\text{j}},{\text{k}},1)} \right),{\text{i}} = 1,2, \ldots ,{\text{S}}$. At the same time, there will be repulsion between bacteria, which prevents the consumption of nearby nutrients by maintaining a certain distance. ${\text{J}}_{{{\text{cc}}}} (\theta ,{\text{P}}({\text{j}},{\text{k}},{\text{l}}))$ said that the combined influence of attraction and repulsion between bacteria is considered at the same time, and its computation formula is as follows:

14 $$ \begin{aligned} {\text{J}}_{{{\text{cc}}}} (\theta ,{\text{P}}({\text{j}},{\text{k}},{\text{l}})) & = \sum\limits_{{{\text{i}} = 1}}^{{\text{s}}} {J_{CC}^{i} (\theta ,\theta^{i} ({\text{j}},{\text{k}},{\text{l}}))} \\ & { = }\sum\limits_{{{\text{i}} = 1}}^{{\text{s}}} {\left[ { - {\text{d}}_{{\text{atact }}} \exp \left( { - {\text{w}}_{{\text{attact }}} \sum\limits_{{{\text{m}} = 1}}^{{\text{p}}} {\left( {\theta_{{\text{m}}} - \theta_{{\text{m}}}^{{\text{i}}} } \right)^{2} } } \right)} \right]} \\ & \quad + \sum\limits_{{{\text{i}} = 1}}^{{\text{s}}} {\left[ {{\text{h}}_{{\text{repenatint }}} \exp \left( { - {\text{w}}_{{\text{repelinat }}} \sum\limits_{{{\text{m}} = 1}}^{{\text{p}}} {\left( {\theta_{{\text{m}}} - \theta_{{\text{m}}}^{{\text{i}}} } \right)^{2} } } \right)} \right]} \\ \end{aligned} $$

where ${\text{P}}({\text{j}},{\text{k}},{\text{l}})$ represents the position of each bacterium in the population $S$ after the $j$ trend operation, the $k$ replication operation, and the $l$ elimination-dispersal operation, $\theta = [\theta_{1} {,} \ldots ,\theta_{D} ]^{T}$ is a point on the optimization domain, $\theta_{m}^{i}$ is the *m* element of the *i* bacteria, dattract represents the amount of attraction released by the bacteria, Wattract is used to measure the width of the attraction signal. hrepenatint indicates the amount of rejection released by the bacteria, and Wrepelinat measures the width of the rejection signal.

However, there are some problems in the original BFO trend activity. First, the bacterial movement step $C(i)$ is given randomly, resulting in a low convergence accuracy of the algorithm. To solve this problem, most scholars choose to design a new step size. Supriyono et al.^50^ developed three types of step size strategies: linear step size, quadratic step size, and exponential step size. Niu et al.^51^ proposed a linear chemotactic decline step and a non-linear chemotactic decline step as well as other types of non-adaptive steps^52–54^.

In addition, the effect of communication between bacterial groups is limited, but the clustering mechanism with complex objective function cannot effectively guide bacterial individuals to the high-nutrient (fitness value) region, resulting in the algorithm often falling into the local optimal value prematurely. To solve this problem, scholars often choose to ignore the original clustering mechanism and combine better communication mechanisms to improve the algorithm. Chen et al.^55^ combined the PSO algorithm to enhance intercellular communication and proposed an adaptive foraging strategy using area-focused search. Wang et al.^56^ also chose to combine the PSO algorithm and Gaussian distribution to adjust the chemotactic activity of the flora and strengthen the ability of information exchange among the populations. Zhao et al.^57^ employed the gravitational mechanism in GSA to improve the ability of information exchange between individuals in the chemotactic step of the BFO algorithm.

The Osprey optimization algorithm was proposed by Mohammad Dehghani and Pavel Trojovsky in 2023 to simulate the predation behavior of Osprey^58^. In the first stage of OOA, the Osprey identifies the position of the fish (fitness value) and performs the arrest (moving in the direction of high fitness and updating the individual position). For each Osprey, the position of the other Osprey with a better target fitness value in the search space is also regarded as the fish school. OOA Phase 2 brings the fish to the appropriate position to feed (moving in a random direction and updating the individual position). Among them, $FP_{i}$ operator with certain clustering and optimal value searching ability in the OOA algorithm, and $x_{i,j}^{P1}$ operator with more optimal positions to update individual positions, can be used to solve the problems in the BFO algorithm. In summary, this study chose to combine the first phase of OOA with BFO chemotaxis to fully improve the performance of the IBFO-A algorithm. The second stage and subsequent elimination-dispersal activities are not selected to be combined with this stage.

The mathematical formula of "flip" movement:

15 $$ FP_{i} = \left\{ {X_{k} |k \in \{ 1,2, \ldots ,N\} \wedge F_{k} < F_{i} } \right\} \cup \left\{ {X_{best \, } } \right\} $$

Formula 15 is used to investigate the search space with a good target value, where FP is a set of i target locations and XB is the best candidate solution.

The mathematical formula of "swimming":

16 $$ x_{i,j}^{P1} = x_{i,j} + r_{i,j} \cdot \left( {SF_{i,j} - I_{i,j} \cdot x_{i,j} } \right) $$

17 $$ X_{i} = \left\{ {\begin{array}{*{20}l} {X_{i}^{P1} ,} \hfill & {F_{i}^{P1} < F_{i} } \hfill \\ {X_{i} ,} \hfill & { \, else \, } \hfill \\ \end{array} } \right. $$

Formula 16 calculates the new position of the individual, and if this new position improves the target fitness value, the previous position is replaced according to Formula 17, where $X_{i}$ is the original position of the individual, $x_{i,j}^{P1}$ is the new position of the individual *i*, $F_{i}^{P1}$ is its objective function value, $SF_{i,j}$ is the candidate solution chosen by the individual i, $r_{i,j}$ is the random number in the interval [0,1], and $I_{i,j}$ is the random number in the set $\left\{ {1,2} \right\}$.

#### Reproductive activity

Reproduction is a fundamental biological behavior observed in various species and is a crucial aspect of life preservation. With each reproductive generation, the search efficiency of the colony improves. It has been mentioned in the conventional BFO algorithm that inferior bacteria should be eliminated and superior bacteria retained for reproduction. The cumulative state of the health fitness value of the i bacteria is represented by $J_{{}}^{i} = \sum\limits_{j = 1}^{Nc + 1} {J\left( {i,j,k,l} \right)}$.

The population of bacteria is divided into two sets based on the accumulation of their health fitness scores: $X_{{{\text{best}}}}^{{}}$, comprising the top-ranked elite individuals with higher cumulative scores and $X_{{{\text{worst}}}}^{{}}$, comprising the lower-ranked inferior individuals with lower cumulative scores. Then, a “genetic” approach was employed to perform reproduction activities following the specific formula below:

18 $$ X_{{{\text{best}}}} = X_{{{\text{best}}}}^{\prime } $$

19 $$ X_{{{\text{co}}}} = X_{{{\text{best}}}}^{\prime } \otimes X_{{{\text{worst}}}} $$

20 $$ X^{\prime } = X_{{{\text{co}}}} \oplus X_{{{\text{best}}}} $$

where $\otimes$ represents a crossover operator employed to perform the "Multi-point crossover"^59^ of the encoding.$\oplus$ denotes the fusion operator. $X_{best}^{\prime }$ represents the reproduction elite individual obtained by $X_{{{\text{best}}}}^{{}}$ after replication and coding. $X_{{{\text{best}}}}^{\prime }$ and $X_{{{\text{worst}}}}^{{}}$ perform crossover operations on their encodings, leading to a single reproductive crossover individual $X_{{{\text{co}}}}^{{}}$. $X^{\prime }$ denotes the new bacterial population obtained by fusing with $X_{{{\text{co}}}}^{{}}$ and $X_{{{\text{best}}}}^{{}}$. This method enables an increase in the chemotactic ability of bacteria with lower cumulative scores, i.e., it enhances the average quality of the entire population while maintaining the original total number of bacterial individuals *S*. Furthermore, it improves the species diversity of the population and prevents the algorithm from becoming trapped in local optima.

#### Elimination-dispersal activity

In this study, the elimination-dispersal activity mechanism was enhanced based on adaptation theory. The bacterial population randomly selects and performs elimination-dispersal activity after each *N*_*r*_ round of reproduction, prompting the bacterial individuals to produce new solutions and conduct a new search for positions, thereby escaping local optima.

The specific definition of the elimination-dispersal function is expressed as follows:

21 $$ P_{{_{ed} }}^{i} = \frac{{\left| {f_{\min }^{{}} } \right| + \left| {(f_{{_{\max } }}^{{}} - f_{i}^{{}} ) * P_{{_{ed} }}^{i - 1} } \right|}}{{\left| {f_{\min }^{{}} } \right| + \left| {(f_{{_{\max } }}^{{}} - f_{\min }^{{}} ) * P_{{_{ed} }}^{i - 1} } \right|}} $$

where $P_{{_{ed} }}^{i}$ is the elimination-dispersal probability at the current moment $P_{{_{ed} }}^{i - 1}$ represents the probability of elimination-dispersal at the previous time.$f_{\min }^{{}}$ represents the worst goal score in history, $f_{{_{\max } }}^{{}}$ represents the best goal score in history, and $f_{i}^{{}}$ represents the current goal score of specific bacteria *i*.

With an increase in the number of iterations, the adaptive elimination-dispersal probability shows a non-linear decreasing trend. At the early stage of iteration, to explore the solution space more widely, a larger elimination-dispersal probability is needed to find other foraging paths. In the later iteration, due to the guidance of the global optimal solution, the algorithm conducts a fine search near the global optimal solution, and the elimination-dispersal probability is reduced. Thus, the local development ability is enhanced, and the bacteria can find the target solution more quickly and accurately. In addition, for formula (21), to determine a better objective function, the population elimination-dispersal probability increases when the current score is close to the lowest score.

### K2 dynamic scoring function

K2 scoring function differs from that of static BN because of the introduction of time information in DBN. A dynamic scoring function is necessary to measure the validity of the network structure. Thus, the K2 dynamic scoring function in IBFO-D is discussed in this section.

First, the initial network $B_{0}$ can be learned from the dataset assuming that the training set consists of $N$ complete sequence samples, where the length of the $l$-th sample is $N_{l}$, and a specific value is assigned to the variable $x^{l} [0], \ldots ,x^{l} [N_{l} ]$. Then, the transitional network $B_{ \to }$ can be learned from the transformed data $N = \sum {N_{l} }$. Considering the definitions of network parameters $t = 1, \cdots ,T$ and sequence samples $\theta$ within the time slice $N$:

22 $$ \begin{array}{*{20}l} {\theta_{i,j,k}^{0} = P\left( {X_{i} [0] = k_{i} |Pa\left( {X_{i} [0] = j_{i} } \right)} \right)} \hfill \\ {\theta_{i,j,k}^{ \to } = P\left( {X_{k} [t] = k_{i} |Pa\left( {X_{k} [t]} \right) = j_{i} } \right)} \hfill \\ \end{array} $$

23 $$ \left\{ {\begin{array}{*{20}l} {N_{i,j,k}^{0} = \sum\limits_{l} I \left( {X_{i} [0] = k_{i} ,Pa\left( {X_{i} [0] = j_{i} ;x^{l} } \right)} \right.} \hfill \\ {N_{{_{i,j,k} }}^{ \to } = \sum\limits_{l} {\sum\limits_{0} I } \left( {X_{i} [t] = k_{i} ,Pa\left( {X_{i} [t]} \right) = j_{i} ;x^{l} } \right)} \hfill \\ \end{array} } \right. $$

where $I$ represents an indicator function, specifically defined as:

24 $$ I = \left\{ {\begin{array}{*{20}l} 1 \hfill & {{\text{if}}\;\left( {X_{i} [t] = k_{i} ,Pa\left( {X_{i} [t]} \right) = j_{i} } \right)} \hfill \\ 0 \hfill & {else} \hfill \\ \end{array} } \right. $$

The joint probability density of DBN is expressed as follows:

25 $$ P_{{{\text{DBN}}}} (x[0],x[1], \ldots ,x[T]) = P_{{B_{0} }} (X[0])\prod\limits_{t = 0}^{T - 1} {P_{{B_{ \to } }} } (x[t + 1]|x[t]) $$

The structure of DBN decomposes the likelihood function distribution into:

26 $$ P(D|G) = \int P \left( {D|G,\theta } \right)P\left( {\theta |G} \right){\text{d}}\theta $$

The first term of the integral is decomposed into the following formula:

27 $$ P\left( {D|G,\theta } \right) = \prod\limits_{i} {\prod\limits_{j} {\prod\limits_{k} {\left( {\theta_{i,j,k}^{0} } \right)^{{N_{i,j,k}^{0} }} } } } \cdot \prod\limits_{i} {\prod\limits_{j} {\prod\limits_{k} {\left( {\theta_{i,j,k} } \right)^{{N_{{_{i,j,k} }}^{ \to } }} } } } $$

The second term of the integral is decomposed into the following formula, assuming that the prior distribution on conditional probabilities is conditionally independent:

28 $$ P\left( {\theta |G} \right) = \prod\limits_{i} {\prod\limits_{j} P } \left( {\theta_{i,j,k}^{0} } \right) \cdot \prod\limits_{i} {\prod\limits_{j} P } \left( {\theta_{i,j,k}^{ \to } } \right) $$

The likelihood function can be rewritten as the product of two integrals by substituting the aforementioned formula:

29 $$ P(D|G) = \prod\limits_{i} {\prod\limits_{j} {\int {\prod\limits_{k} {\left( {\theta_{i,j,k}^{0} } \right)^{{N_{i,j,k}^{0} }} } } \times P\left( {\theta_{i,j,k}^{0} } \right) \times {\text{d}}\theta_{i,j,k}^{0} } } $$

The likelihood function $P(D|G)$ can be further expressed as a product of $K2_{0}$ and $K2_{ \to }$ given the hyperparameter $N_{i,j,k}^{0} N_{{_{i,j,k} }}^{ \to }$ and complete data, and with the parameter prior following a Dirichlet distribution:

30 $$ P(D|G) = \prod\limits_{i = 1}^{n} {\prod\limits_{j = 1}^{{q_{i} }} {\frac{{(r_{i} - 1)!}}{{(N_{{_{i,j} }}^{0} + r_{i} - 1)!}}} } \cdot \prod\limits_{k = 1}^{{r_{i} }} {N_{{_{i,j,k} }}^{ \to } !} $$

where n denotes the number of variable samples, $q_{i}$ denotes the number of parent nodes for $X_{i}$, $r_{i}$ denotes the number of possible values for $X_{i}$, $N_{{_{i,j} }}^{0}$ denotes the number of samples, and $N_{{_{i,j,k} }}^{ \to }$ denotes the total number of samples.

This study selects the logarithm of the likelihood function to minimize in practical applications. The final expression of the dynamic K2 scoring formula is as follows:

31 $$ \begin{aligned} Score(G|D) & = K2_{0} (G|D) + K2_{ \to } (G|D) \\ & { = }\sum\limits_{i = 1}^{n} {q_{i} } \cdot \log \left( {r_{i} - 1} \right)! - \sum\limits_{j = 1}^{{q_{i} }} {\log } \left( {N_{{_{i,j} }}^{0} + r_{i} - 1} \right)! + \sum\limits_{k = 1}^{{r_{i} }} {\log } \left( {N_{{_{i,j,k} }}^{ \to } } \right)! \\ \end{aligned} $$

### IBFO-D optimization algorithm

#### Initialization

In Swarm intelligent optimization algorithms, encoding approaches to generate abstract structures and the concretization of the optimization process are crucial elements. In this study, the network structure is represented using an adjacency matrix $A = (a_{ij} )$ with $n \times n$ dimensions. The directed edge from node $i$ to node $j$ is represented by $a_{ij} = 1$, whereas the absence of a connection between node $i$ and node $j$ is denoted by $a_{ij} = 0$.

32 $$ a_{ij} = \left\{ {\begin{array}{*{20}l} 1 \hfill & { \, i\;{\text{ is}}\;{\text{ a}}\;{\text{ parent}}\;{\text{ of}}\; \, j} \hfill \\ 0 \hfill & {{\text{ no}}\;{\text{ edges}}\;{\text{ or}}\;{\text{ deleted}}\;{\text{ edges }}} \hfill \\ \end{array} } \right. $$

**Figure 3:**
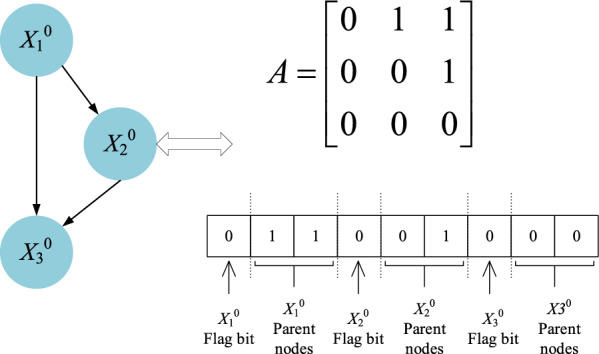
Initial network $$B_{0}$$.

**Figure 4:**
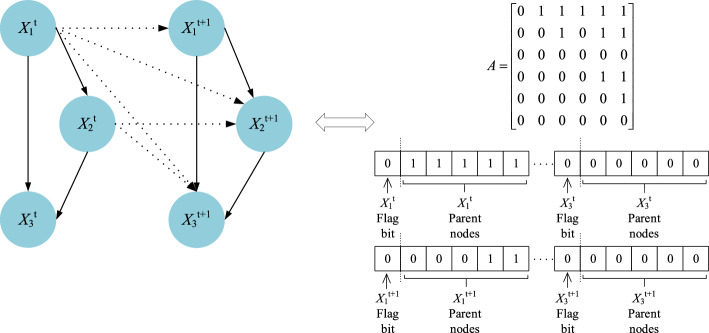
Transfer network $$B_{ \to }$$.

The specific process first initializes an empty adjacency matrix, develops and explores the search space through bacteria in the direction of high fitness, and constantly updates the location if and only if the new location has a higher score. Then, the network graph structure is updated by directional rules, and the process is repeated until the network structure with the highest K2 score in the time slice $t$ is found, and the optimization iteration of the $t + 1$ time slice is started. The DBN structure is represented as a DAG. Thus, it is crucial to consider their validity when constructing the initial network. In other words, in the searched DBN, the network structure should not contain any cycles or bidirectional edges. Reflected in the adjacency matrix, this indicates that the nodes in the matrix should not form cycles and that elements symmetrically located about the diagonal should not be to 1. Figures 3 and 4 illustrate examples of generating initial network $B_{0}$ and transfer network $B_{ \to }$ structural adjacency matrices, respectively. Node labels can be simplified to further reduce the search space, leading to $B_{0} = 11|01|00$ and $B_{ \to } = 00110|00011|00001|00010|00001|00000$.

#### Network structure learning

IBFO-D In the framework of the IBFO-A algorithm, the state relationship between nodes in the DBN was considered. Restrictions were imposed on the tendency directions of the bacteria, such as $N_{S}$ and $N_{C}$, considering the state relationships between nodes in the DBN. Consequently, three edge orientation rules, namely “add edge,” “remove edge,” and “reverse edge,” were designed:

1. Add Edge: Given a collection of nonempty nodes $X = \left\{ {x_{1} ,x_{2} , \ldots ,x_{i} ,x_{i + 1} , \ldots ,x_{j} |x_{i} \in G,x_{i} \notin \prod {(x_{j} )} } \right\}$, if $e_{ij} = x_{i} \to x_{j}$ is added and $e_{ij} \in G$ holds, then $G^{\prime} \in G \cup (e_{ij} )$;
2. Remove Edge: Given a directed edge set $E = \left\{ {e_{ij} = x_{i} \to x_{j} |x_{i} \in \prod {(x_{j} )} ,i = 1, \cdots ,n} \right\}$, if $e_{ij}$ is removed, then $G^{\prime} \in G\backslash (e_{ij} )$ $X = \left\{ {x_{1} ,x_{2} , \ldots ,x_{i} ,x_{i + 1} , \ldots ,x_{j} |x_{i} \in G,x_{i} \notin \prod {(x_{j} )} } \right\}``$ and $e_{ij} = x_{i} \to x_{j}$ are removed;
3. Reverse Edge: Given a directed edge set $E = \left\{ {e_{ij} = x_{i} \to x_{j} |x_{i} \in \prod {(x_{j} )} ,i = 1, \ldots ,n} \right\}$, if $e_{ij}$ is removed, $e_{ji} = x_{j} \to x_{i}$ is added, and $e_{ji} \in G$ holds, then $G^{\prime } \in G\backslash (e_{ij} ) \cup (e_{ji} )$.

The chemotactic activity process continued until the bacteria reached a fixed position and no longer moved or had moved the maximum number of chemotaxis, which corresponded to finding the network structure with the highest K2 score in the DBN network or reaching the upper limit of search iterations.

To choose healthy bacteria, it is essential to assess the health level of each bacterium. This assessment involves computing the sum of the fitness values of the chemotaxis steps. A higher cumulative value signifies that maximum nutrition has been obtained, making it more suitable for reproduction. In this study, the step fitness value of bacteria is defined based on the dynamic scoring function in DBN structure learning. Specifically, the health score for the i bacterium is expressed as follows:

33 $$ HS_{t}^{i} \, = K2_{0(G|D)} (i,j,k,l) + \sum\limits_{{j_{t} = 1}}^{{N_{c} }} {K2_{ \to (G|D)} (i_{t} ,j_{t} ,k_{t} ,l_{t} )} $$

$K2_{0(G|D)} (i,j,k,l)$ is defined as the prior fitness value function for the $i$ bacterium during the $j$ chemotaxis, $k$ reproduction, and $l$ elimination-dispersal when generating the initial network. $K2_{ \to (G|D)} (i_{t} ,j_{t} ,k_{t} ,l_{t} )$ is the fitness value function for subsequent transition networks. Health function assesses the accumulated K2 score for individual bacteria throughout the entire process of chemotaxis operations.

To determine the global optimal network structure, the specific definition of the elimination-dispersal function is expressed as follows:

34 $$ P_{{_{ed} }}^{t} = \frac{{\left| {HS_{\min }^{{}} } \right| + \left| {(HS_{{_{\max } }}^{{}} - HS_{t}^{i} ) * P_{{_{ed} }}^{t - 1} } \right|}}{{\left| {HS_{\min }^{{}} } \right| + \left| {(HS_{{_{\max } }}^{{}} - HS_{\min }^{{}} ) * P_{{_{ed} }}^{t - 1} } \right|}} $$

where $P_{{_{ed} }}^{t}$ is the elimination-dispersal probability at the current moment. $P_{{_{ed} }}^{t - 1}$ is the elimination-dispersal probability of the previous moment. $HS_{\min }^{{}}$ is the lowest historical health score, $HS_{{_{\max } }}^{{}}$ is the highest historical health score, and $HS_{t}^{i}$ is the current specific health score of bacterium *i*.

#### Algorithm description

The IBFO-D Algorithm proposed in this study is shown in Algorithm 1. The whole DBN structure learning process is summarized as follows: the algorithm starts from the initialization of network parameters, randomly generates the initial DAG population, and finds the high-quality network structure through the chemotactic activity formula (15–17). At the same time, the driving force of DBN local optimization is generated according to three operators. The fitness value of each bacterium, namely the K2 score, was calculated, and the cumulative value $HS_{t}^{i}$ was recorded as a health score. By selecting elite individuals with high $HS_{t}^{i}$, the average optimization ability of the bacterial population was updated according to the formula (18–20) to improve the convergence speed, while preserving certain species diversity to prevent falling into local optimality. According to formula (34), determine whether the bacterial individual generates a new solution and re-searches. According to the above optimization steps, as well as the dynamic K2 scoring measures and constraints, until a high-score network structure matching the data set is searched.

**Algorithm 1:**
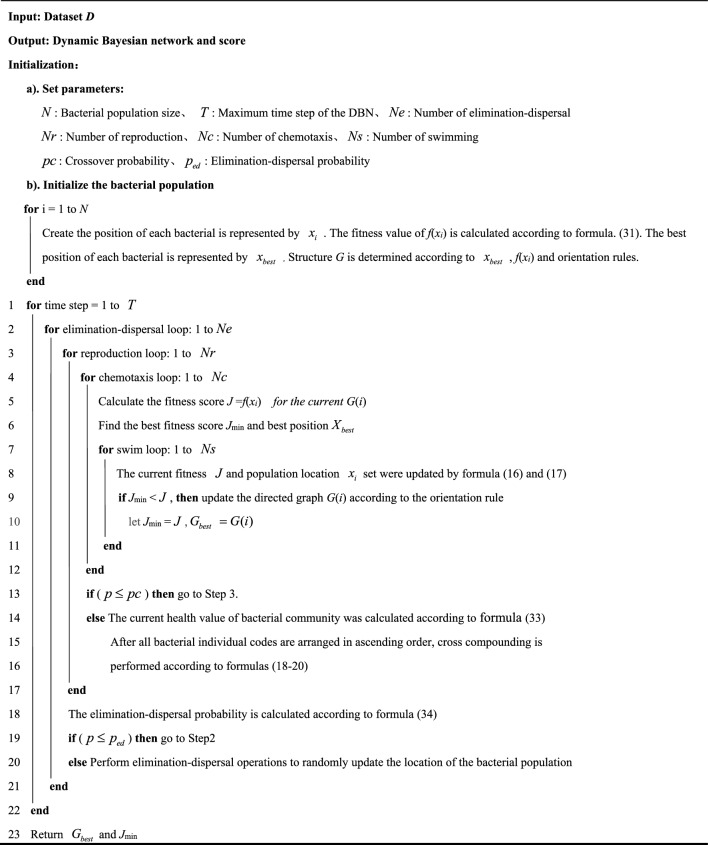
IBFO-D

## Experimental section

### Experimental preparation

In the test experiment, we mainly focus on the optimization performance of the proposed method and its learning effect on the DBN model structure, and do not deny the validity and novelty of other optimization algorithms and their modeling in the corresponding domain. The parameter values of each algorithm used in the experiment are shown in Table 1.

**Table 1:** Parameter settings for the algorithms used.

Method	Population size	Maximum generation	Other parameters
IBFO-A	60	1000	$r = 0.86$; $P_{ed} = 0.8$; $pc = 0.2$
BFO	60	1000	$P_{ed} = 0.8$; $\Delta \in \left[ { - 1, \, 1} \right]$
OOA	60	1000	$r \in \left[ {0, \, 1} \right]$; $I = \{ 1,2\}$
GA	60	1000	$pc = 0.8$; $pm = 0.05$
GWO	60	1000	$a \in [2,0]$; $\left| {r_{1} } \right| \in [0,1]$; $\left| {r_{2} } \right| \in [0,1]$
PSO	60	1000	$w_{\max } = 0.9$; $w_{\min } = 0.6$; $v_{\max } = 6$; $c_{1} = 2$; $c_{2} = 2$
BWO	60	1000	$r_{1} \in \left( {0,1} \right)$; $r_{2} \in \left( {0,1} \right)$; $\beta = 1.5$
DBO	60	1000	$k \in \left( {0,0.2} \right]$; $b \in \left( {0,1} \right)$
COA	60	1000	$temp \, = \, rand*15 + 20$
GO	60	1000	${\text{pro}} \in \left( {0,1} \right)$; ${\text{rnd}} \in \left( {0,1} \right)$; ${\text{coe}} \in \left( {0,1} \right)$
AWPSO	60	1000	$w_{\max } = 0.9$; $w_{\min } = 0.4$; $v_{\max } = 0.5$; $v_{\min } = - 0.5$
ChaoticBFO	60	1000	$\mu = 0.8$; $P_{ed} = 0.8$; $\Delta \in \left[ { - 1, \, 1} \right]$

To test the optimization performance of the proposed algorithm, Firstly, we use IBFO-A and seven other optimization algorithms to perform comparative test experiments on 10 different benchmark functions. The reference function comes from CEC2005^60^, including the multi-peak function, single-peak function, and fixed-dimensional multi-peak function, which is used to test the convergence speed, accuracy, effectiveness, and global search ability of the algorithm. The specific reference function is shown in Table 2. Then we select three kinds of hyperparameters for parameter sensitivity analysis to test the stability of the algorithm.

**Table 2:** CEC2005 benchmark functions used in the experimental study.

Test function	Category	$D$	$S$	$f_{\min }$
$f_{1} (x) = \sum\limits_{i = 1}^{n} {x_{i}^{2} }$	Unimodal test functions	30	$\left[ { - 100,100} \right]^{n}$	0
$f_{2} (x) = \sum\limits_{i = 1}^{n} {\left| {x_{i} } \right|} + \prod\limits_{i = 1}^{n} {\left| {x_{i} } \right|}$		30	$\left[ { - 10,10} \right]^{n}$	0
$f_{3} (x) = \sum\limits_{i = 1}^{n} {\left( {\sum\limits_{j = 1}^{i} {x_{j} } } \right)^{2} }$		30	$\left[ { - 100,100} \right]^{n}$	0
$f_{4} (x) = \sum\limits_{i = 1}^{n} {\left[ {x_{i}^{2} - 10\cos \left( {2\pi x_{i} } \right) + 10} \right]}$	Multimodal test functions	30	$\left[ { - 5.12,5.12} \right]^{n}$	0
$f_{5} (x) = \frac{1}{4000}\sum\limits_{i = 1}^{n} {x_{i}^{2} } - \prod\limits_{i = 1}^{n} {\cos } \left( {\frac{{x_{i} }}{\sqrt i }} \right) + 1$		30	$\left[ { - 600,600} \right]^{n}$	0
$$\begin{aligned} f_{6} (x) & = 0.1\left\{ {\sin^{2} \left( {3\pi x_{1} } \right) + \sum\limits_{i = 1}^{n - 1} {\left( {x_{i} - 1} \right)^{2} } \left[ {1 + \sin^{2} \left( {3\pi x_{i + 1} } \right)} \right]} \right. \\ & \quad \left. { + \left( {x_{n} - 1} \right)\left[ {1 + \sin^{2} \left( {2\pi x_{n} } \right)} \right]} \right\} + \sum\limits_{i = 1}^{n} u \left( {x_{i} ,5,100,4} \right) \\ \end{aligned}$$		30	$\left[ { - 50,50} \right]^{n}$	0
$f_{7} (x) = \sum\limits_{i = 1}^{11} {\left[ {a_{i} - \frac{{x_{1} \left( {b_{i}^{2} + b_{i} x_{2} } \right)}}{{b_{i}^{2} + b_{i} x_{3} + x_{4} }}} \right]^{2} }$	Multimodal test functions with fix dimension	4	$\left[ { - 5,5} \right]^{n}$	3.075e−4
$$\begin{aligned} f_{8} (x) & = \left[ {1 + \left( {x_{1}^{{}} + x_{2} + 1} \right)^{2} \left( {19 - 14x_{1} + 3x_{1}^{2} - 14x_{2} } \right.} \right. \\ & \quad \left. {\left. { + 6x_{1} x_{2} + 3x_{2}^{2} } \right)} \right] \times \left[ {30 + \left( {2x_{1} - 3x_{2} } \right)^{2} \left( {18 - 32x_{1} } \right.} \right. \\ & \quad \left. {\left. { + 12x_{1}^{2} + 48x_{2} - 36x_{1} x_{2} + 27x_{2}^{2} } \right)} \right] \\ \end{aligned}$$		2	$\left[ { - 2,2} \right]^{n}$	3
$f_{9} (x) = - \sum\limits_{i = 1}^{4} {c_{i} } \exp \left[ { - \sum\limits_{j = 1}^{6} {a_{ij} } \left( {x_{j} - p_{ij} } \right)^{2} } \right]$		6	$\left[ {0,1} \right]^{n}$	− 3.32
$f_{10} (x) = - \sum\limits_{i = 1}^{10} {\left[ {\left( {x - a_{i} } \right)\left( {x - a_{i} } \right)^{T} + c_{i} } \right]^{ - 1} }$		4	$\left[ {0,10} \right]^{n}$	− 10

where $D$ is the dimension of the function, $f_{\min }$ is the minimum value of the function, and search range $S \subseteq R^{n}$.

Secondly, We conducted several more sets of comparative experiments of optimization algorithms. These include the eight optimization algorithms mentioned above and some novel optimization algorithms as well as improved optimization algorithms. The benchmark function for the simulation experiments is from CEC2019, which is a popular benchmark function that is quite effective in testing the performance of optimization algorithms. Then we use 2 sets of real-world engineering optimization problems to test the optimization performance of the algorithms from multiple perspectives. The CEC2019 specific reference function is shown in Table 3.

**Table 3:** CEC2019 benchmark functions used in the experimental study.

Test function	Description	$D$	$S$	$f_{\min }$
F1	Storn's Chebyshev polynomial fitting problem	9	$\left[ { - 8192,8192} \right]$	1
F2	Inverse Hilbert matrix problem	16	$\left[ { - 16384,16384} \right]$	1
F3	Lennard–Jones minimum energy cluster	18	$\left[ { - 4,4} \right]$	1
F4	Rastrigin's function	10	$\left[ { - 100,100} \right]$	1
F5	Grienwank's function	10	$\left[ { - 100,100} \right]$	1
F6	Weierstrass funetion	10	$\left[ { - 100,100} \right]$	1
F7	Modified Schwefel's function	10	$\left[ { - 100,100} \right]$	1
F8	Expanded Schaffer's F6 function	10	$\left[ { - 100,100} \right]$	1
F9	Happy Cat function	10	$\left[ { - 100,100} \right]$	1
F10	Ackley function	10	$\left[ { - 100,100} \right]$	1

Finally, for the IBFO-D algorithm network learning performance test, various dynamic benchmark network experiments were selected. These dynamic benchmark networks were derived from two well-known static BN networks: the Asia and Alarm networks. These 2T-BN networks comprise two time slices and include an initial network represented as $B_{0}$ and a transition network represented as $B_{ \to }$. Benchmark network data can be obtained from the provided Supplementary URL online.

The experimental setup comprised the following environment: Windows 11 operating system, MATLAB and Python programming language, 32.0 GB RAM, an Intel Core i7-12700 K CPU running at 5.0 GHz, and an NVIDIA GeForce RTX 3080Ti graphics card.

### Experimental results and analysis of IBFO-A compared with other SI algorithms

#### Comparison and analysis of eight optimization algorithms in CEC2005 benchmark functions

The experimental design of algorithm optimization performance comparison is as follows: The optimization algorithm in IBFO-A is compared with three original optimization algorithms, BFO, OOA, and GA; two classical optimization algorithms, GWO and PSO, two recent advanced optimization algorithms BWO^61^ and DBO^62^, and a total of eight optimization algorithms are compared and tested on 10 groups of different types of benchmark functions. We uniformly set experimental parameters for all optimization algorithms, in which the population size is set to $N = 60$, the maximum number of iterations $T = 1000$, and the upper bound $ub$, lower bound $lb$, dimension $D$, and optimal value $f_{\min }$ of different test functions are set as shown in Table 2. We present 10 sets of optimization convergence curves, specific scores, and running timelines, and record the average score ranking (ASR) and average run-time ranking (ATR) of the algorithm that runs 30 times independently (if both algorithms converge to the optimal value, the ASR is determined by the number of iterations).

The simulation results in Table 4 show that IBFO-A can converge to the optimal value for 6 of the 10 benchmark functions. In unimodal and multimodal functions, IBFO-A converges directly to the optimal value 0 on the F1, F2, F3, F4, and F5 functions. In addition, it can converge directly to the optimal value 3 on the F8 function, and it is also very close to the theoretical optimal value in other fixed-dimensional multi-peak test functions, which shows that it has good global optimization ability.

**Table 4:** Comparison results of IBFO-A, BFO, OOA, GA, GWO, PSO, BWO, and DBO on the 10 benchmark functions.

	IBFO-A		BFO		OOA		GA		GWO		PSO		BWO		DBO	
	Score	Time	Score	Time	Score	Time	Score	Time	Score	Time	Score	Time	Score	Time	Score	Time
F1	0	0.34518	3.8826e−4	0.304	0	0.37524	7.2368e−03	0.35948	1.1303e−152	0.27802	1.1153e−10	0.11689	0	0.49239	0	0.39853
F2	0	0.35709	1.3612	0.26638	0	0.37985	0.025851	0.38608	3.2631e−84	0.27342	3.9462e−06	0.13724	1.0557e−245	0.60831	5.7691e−179	0.4057
F3	0	0.50364	0.19572	0.26274	0	0.74867	3692.011	0.605	7.4765e−78	0.40169	7.6957e−05	0.2619	0	0.65241	0	0.80969
F4	0	0.306	0	0.24253	0	0.31499	3.0364	0.35543	0	0.29823	1.9909	0.13859	0	0.46038	0	0.38498
F5	0	0.37711	0	0.25357	0	0.45344	1.0136	0.40474	0	0.32898	0.15506	0.18492	0	0.52517	0	0.43327
F6	7.1435e−11	0.88702	1.3959e−05	0.25567	4.7116e−32	1.486	9.3882e−05	0.90122	4.7202e−08	0.73673	6.992e−13	0.61882	4.9019e−07	1.0446	4.7358e−32	0.90217
F7	3.0753e−04	0.37903	2.0981e−3	0.19671	1.1452e−3	0.39969	2.2021e−2	0.39899	2.0363e−2	0.23916	9.8938e−4	0.09926	3.0749e−04	0.45177	1.2232e−3	0.4013
F8	3	0.30494	3	0.24963	3	0.31474	3	0.33	3	0.16243	3	0.060188	3.2	0.36315	3	0.30783
F9	− 3.2374	0.42087	− 3.0711	0.39666	− 2.993	0.41104	− 1.9379	0.38357	− 3.203	0.25917	− 3.2031	0.12689	− 3.3136	0.45415	− 3.322	0.36787
F10	− 10.5364	0.58074	− 4.0465	0.29268	− 10.5363	0.69076	− 2.2012	0.5037	− 10.5363	0.35811	− 10.5364	0.26637	− 10.5364	0.59392	− 5.1756	0.50704
ASR	1		6		2		8		4		7		5		3	
ATR	4		2		7		5		3		1		8		6	

**Figure 5:**
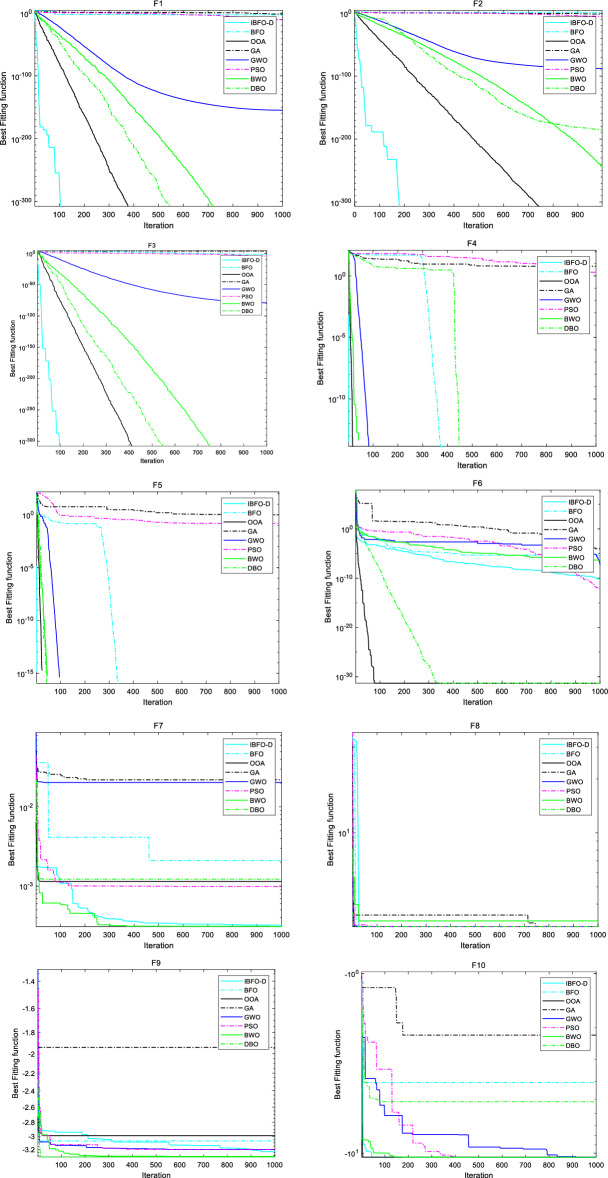
Comparison of convergence curves of 8 optimization algorithms.

Here, we choose the original BFO, OOA, and GA algorithms as reference objects. According to the analysis in Fig. 5, the GA and BFO algorithms perform poorly on F1, F2, and F3 unimodal functions, BFO improves somewhat on multi-modal functions F4 and F5, and the OOA algorithm performs better. IBFO-A can stably converge to the optimal with less than half of the OOA iterations. GA still performs poorly in F7 and F9 functions of fixed dimension, and the convergence values of OOA and BFO are also different from the theoretical optimal values. Compared with the IBFO-A algorithm, the performance of the IBFO-A algorithm is competitive. From the experimental data in Table 4 and the convergence curve in Fig. 5, except for poor performance on the F6 generalized penalized function, compared with the other seven algorithms, IBFO-A exhibits the best performance in the seven function scoring tests, and ASR ranks first. This shows that the IBFO-A algorithm has good convergence speed and accuracy, and proves that the improved chemotactic step and the replication step using cross strategy can avoid falling into the local optimal solution and enhance the local search ability of the algorithm. In addition, the ATR of the IBFO-A algorithm ranks fourth. From the perspective of algorithm time complexity, the time complexity of IBFO-A and BFO is $O(n)$, whereas that of OOA is $O(n^{2} )$. Therefore, the computation time of IBFO-A is better than that of OOA. However, due to the extra computing steps, it consumes more computing time than the classical BFO, PSO, and GWO, which is also a limitation of the algorithm in this study.

#### Sensitivity analysis

**Figure 6:**
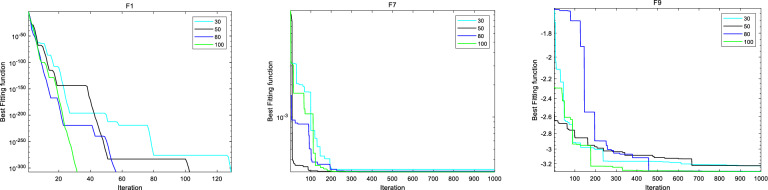
Sensitivity analysis of IBFO-A to parameter *N.*

**Figure 7:**
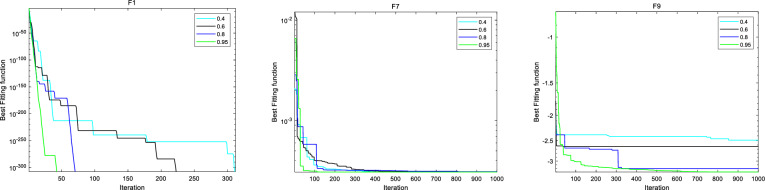
Sensitivity analysis of IBFO-A to parameter *P*_*ed*_*.*

**Figure 8:**
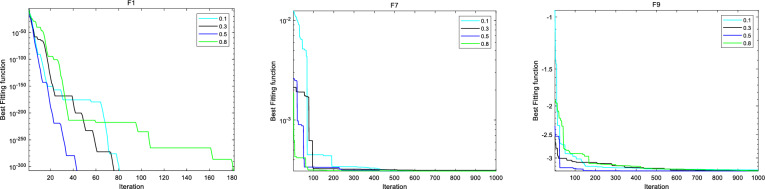
Sensitivity analysis of IBFO-A to parameter *pc.*

This section discusses the hyperparameter sensitivity analysis of IBFO-A algorithm. We selected three hyperparameters that mainly affect the optimization performance of IBFO-A algorithm, including population size *N*, elimination-dispersal probability $P_{{_{ed} }}^{{}}$, and crossover probability $pc$. We set four different parameter values for each hyperparameter to be discussed, and use the IBFO-A algorithm to optimize several typical test functions of CEC2005 under these parameter settings. The specific parameter values and results are shown in Figs. 6, 7, 8 and Table 5.

**Table 5:** Parameter sensitivity comparison experiment.

	F1		F7		F9	
	Score	Time	Score	Time	Score	Time
N						
30	0	0.26881	3.2398e−4	0.34955	− 3.2291	0.39857
50	0	0.28275	3.0818e−4	0.34564	− 3.2296	0.40963
80	0	0.33146	3.1231e−4	0.37591	− 3.3105	0.47671
100	0	0.35398	3.0753e−4	0.37827	− 3.3136	0.51034
*P*_*ed*_						
0.4	0	0.43908	3.0939e−4	0.43906	− 2.4921	0.40801
0.6	0	0.4155	3.0869e−4	0.41504	− 2.6352	0.37525
0.8	0	0.34138	3.0749e−4	0.39222	− 3.2036	0.39457
0.95	0	0.36583	3.0749e−4	0.39482	− 3.3043	0.39887
*pc*						
0.1	0	0.33018	3.0956e−4	0.32449	− 3.2927	0.40793
0.3	0	0.34028	3.1217e−4	0.34316	− 3.3085	0.40045
0.5	0	0.34936	3.0761e−4	0.34173	− 3.3193	0.43215
0.8	0	0.35788	3.0766e−4	0.39245	− 3.3164	0.42945

From the sensitivity analysis of IBFO-A to hyperparameter $N$, it can be seen that with the increase of population size, the probability of finding the global optimal solution will increase, thus improving the optimization performance of the algorithm. However, large population sizes can also lead to increased time costs. $P_{{_{ed} }}^{{}}$ determines the probability of initial elimination-dispersal occurrence of individual bacteria. According to the sensitivity analysis of IBFOA to hyperparameter $P_{{_{ed} }}^{{}}$, high $P_{{_{ed} }}^{{}}$ parameter value enables bacteria to explore the solution space more extensively in the early stage of iteration, which increases the possibility of quickly searching for the global optimal solution. In addition, the improved adaptive activity solves the problem that excessive elimination-dispersal probability in the late iteration will lead to frequent update of bacterial colony location, which makes it difficult to conduct fine search near the optimal solution. In addition, it can be found that IBFO-A algorithm can also search the global optimal solution when $P_{{_{ed} }}^{{}}$ parameter value is low, but it needs more iterations and time cost. From the sensitivity analysis of IBFO-A to hyperparameter $pc$, it can be seen that the higher the probability of $pc$, the more the coding composition of the individual in the flora is affected by other individuals, thus improving the optimization ability of the flora. However, even if IBFO-A uses the improved reproductive activity, too high a $pc$ probability may still lead to a decrease in bacterial diversity, putting the algorithm at risk of falling into a local optimal solution. Finally, according to the optimization results and running time analysis, IBFO-A algorithm is not sensitive to the change of hyperparameters within a reasonable range.

#### Comparison and analysis of twelve optimization algorithms in CEC2019 benchmark functions

**Figure 9:**
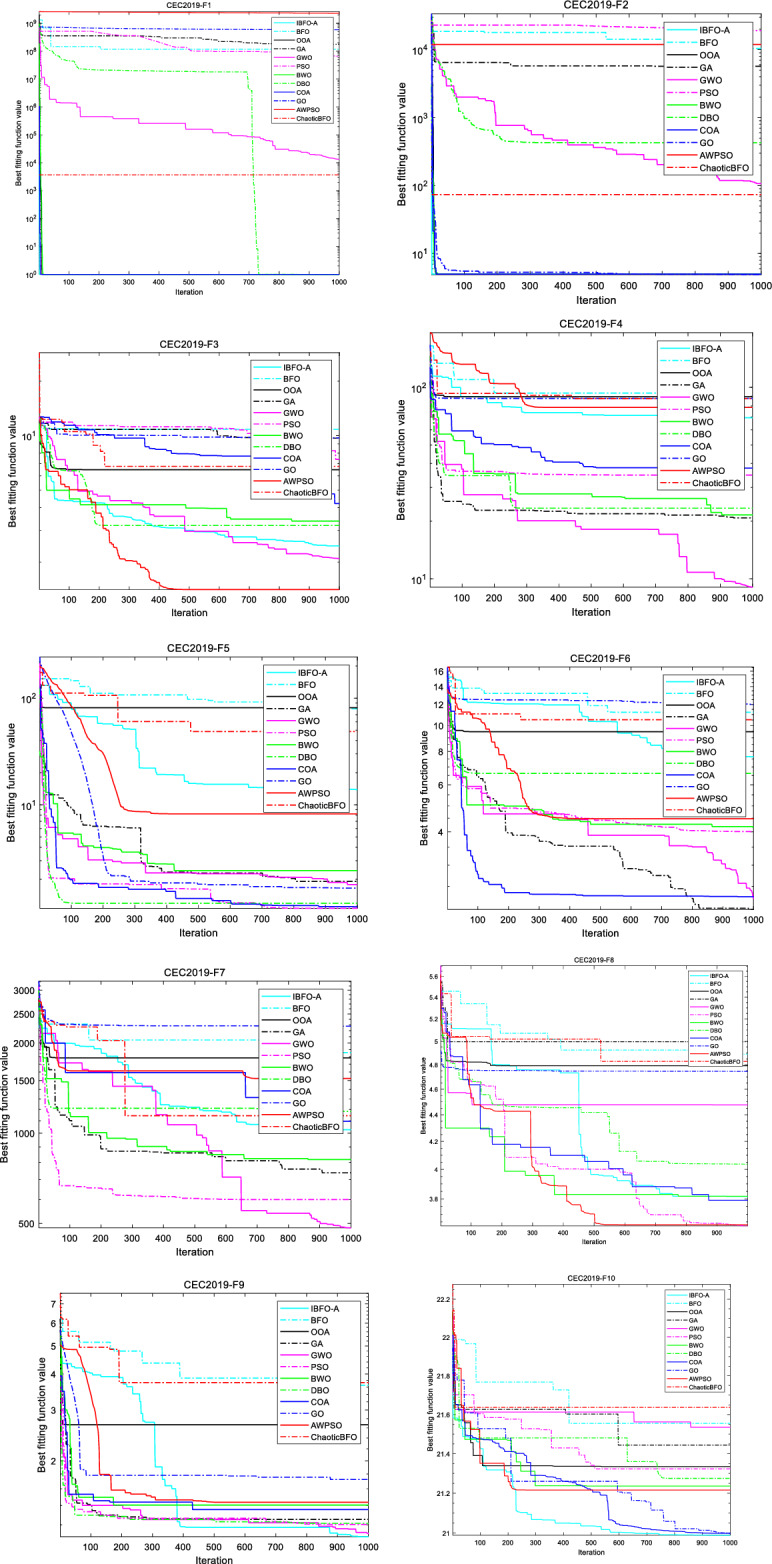
Comparison of convergence curves of 12 optimization algorithms.

The experimental design of algorithm optimization performance comparison is as follows: In addition to the eight optimization algorithms mentioned above, including IBFO-A, Two novel and improved optimization algorithms for PSO and BFO :AWPSO^25^ and ChaoticBFO^26^, as well as the two latest advanced optimization algorithms COA^63^ and GO^64^, a total of 12 optimization algorithms were compared in 10 sets of different types of advanced benchmark functions in CEC2019. We set experimental parameters uniformly for all optimization algorithms. The specific 10 groups of optimization convergence curves, scores and running schedules are shown in Fig. 9 and Table 6.

**Table 6:** Comparison results of IBFO-A, BFO, OOA, GA, GWO, PSO, BWO, DBO, COA, GO, AWPSO, and ChaoticBFO on the CEC2019 benchmark functions.

	IBFO-A				BFO				OOA				GA				GWO				PSO			
	Score	Time	ASR|ATR		Score	Time	ASR|ATR		Score	Time	ASR|ATR		Score	Time	ASR|ATR		Score	Time	ASR|ATR		Score	Time	ASR|ATR	
F1	1	0.51471	1	9	1.1779e+8	0.13126	12	1	1	0.66938	10	11	1.7816e+8	0.57757	7	8	1.3322e+4	0.61903	3	6	6.5303e+7	1.0164	5	12
F2	4.9857	0.43262			1.0458e+4	0.20157			5	0.43205			5.6961e+3	0.51007			1.0655e+2	0.41483			1.9105e+4	0.59639		
F3	2.4498	0.31644			10.837	0.085054			6.4824	0.4427			9.6893	0.25438			2.0939	0.33925			7.3875	0.47824		
F4	69.4439	0.37462			78.9059	0.12656			89.3174	0.52289			20.7976	0.26875			9.0293	0.30214			34.8285	0.52836		
F5	13.8409	0.35037			79.3276	0.16411			81.1214	0.55487			1.9168	0.49689			1.7881	0.35106			1.0714	0.49645		
F6	7.6439	3.7762			11.216	0.72694			9.4888	4.6178			2.046	2.4695			2.2431	2.3497			4.0063	4.6507		
F7	1.0269e+3	0.41371			1.8581e+3	0.15723			1.7834e+3	0.55431			7.3754e+2	0.37282			4.8324e+2	0.58203			6.0044e+2	0.80284		
F8	3.8167	0.33895			4.8899	0.088168			4.7926	0.3799			4.9967	0.27517			4.4762	0.24203			3.6252	0.42283		
F9	1.0912	0.36772			3.6541	0.081567			2.6682	0.4817			1.2533	0.25649			1.1158	0.22874			1.2037	0.35686		
F10	20.9891	0.33267			21.555	0.16646			21.3348	0.46354			21.4439	0.56025			21.535	0.25705			21.3227	0.40833		

	BWO				DBO				COA				GO				AWPSO				ChaoticBFO			
	Score	Time	ASR|ATR		Score	Time	ASR|ATR		Score	Time	ASR|ATR		Score	Time	ASR|ATR		Score	Time	ASR|ATR		Score	Time	ASR|ATR	
F1	1	0.48294	4	10	1	0.45176	6	7	1	0.43022	2	4	5.9018e+8	0.4726	9	5	2.31e+9	0.36643	8	3	3.6731e+3	0.17772	11	2
F2	5	0.62992			4.2723e+2	0.44874			5	0.42175			4.998	0.60817			1.1964e+4	0.27733			73.933	0.17162		
F3	3.3701	0.40246			3.1877	0.27811			4.213	0.2029			9.7112	0.28002			1.4091	0.19957			6.7682	0.1404		
F4	21.5619	0.38112			23.3781	0.29205			37.838	0.37738			87.5607	0.28915			78.6063	0.4423			87.3588	0.21643		
F5	2.4201	0.42112			1.1945	0.30336			1.1084	0.33756			1.6568	0.34559			8.2245	0.20874			48.5569	0.13086		
F6	4.1681	2.9611			6.6196	2.4659			2.2831	3.8721			12.0047	2.4237			4.4743	2.3223			10.5205	0.76085		
F7	8.1706e+2	0.63241			1.182e+3	0.41399			1.095e+3	0.3335			2.2759e+3	0.3119			1.5242e+3	0.23214			1.1435e+3	0.13789		
F8	3.8176	0.50389			4.0369	0.36719			3.7907	0.33956			4.7452	0.35088			3.6329	0.21435			4.8279	0.17259		
F9	1.405	0.44			1.2145	0.44716			1.3554	0.4415			1.7225	0.28468			1.4362	0.19956			3.7324	0.19608		
F10	21.236	0.37332			21.2753	0.30392			20.999	0.25123			21.0001	0.29586			21.2158	0.21668			21.6367	0.13173		

According to the experimental data in convergence diagram 9 and Table 6, compared with other 11 algorithms, IBFO-A has the best performance in four function scoring tests, and ASR ranks first, indicating that IBFO-A has good optimization performance. IBFO-A performs well in F1 and F10 test functions designed for single-objective real parameter optimization, demonstrating the IBFO-A algorithm's good performance in the global search for the best solution. It also performs well in the two high-dimensional test functions F2 and F3, This shows that the improved chemotactic activity and replication activity achieve a harmonious equilibrium between exploration and exploitation. It makes IBFO-A algorithm have better searching ability in test functions of different dimensions, and can be used to optimize DBN structure model. In addition, the basic algorithm BFO and another improved algorithm, ChaoticBFO, are also competitive in F1 and F2 compared with other test functions, but their performance is slightly inferior in F3 test functions, which may be because the random elimination-dispersal activity they use is difficult to escape the local optimal solution under high-dimensional functions. For the test function of fixed-dimensional multi-modal and multi-objective optimization, IBFO-A ranks among the best in F7-F9 and performs well in F4-F6, indicating that IBFO-A algorithm can be applied to multimodal and multi-objective optimization problems. In addition, the ATR of IBFO-A algorithm ranks 9th, indicating that the running time of IBFO-A algorithm has increased in complex optimization problems.

#### Two real-world engineering optimization problems

In this section, we use two different real-world engineering optimization problems to evaluate the model optimization capabilities of the IBFO-A algorithm, where each set of algorithms is run independently 50 times. Two kinds of engineering optimization problem parameter selection are shown in Table 7.

**Table 7:** Two kinds of engineering optimization problem parameter selection.

Test function	Subject to	$S$
$\min f(x) = \left( {x_{3} + 2} \right)x_{2} x_{1}^{2}$	$q_{1} (x) = 1 - \frac{{x_{2}^{3} x_{3} }}{{71785x_{1}^{4} }} \le 0$	$2 \le x_{1} \le 15$ $0.25 \le x_{2} \le 1.3$ $0.05 \le x_{3} \le 2$
	$$q_{2} ({\text{x}}) = \frac{{4{\text{x}}_{2}^{2} - {\text{x}}_{1} {\text{x}}_{2} }}{{1256\left( {{\text{x}}_{2} {\text{x}}_{1}^{3} - {\text{x}}_{1}^{4} } \right)}} + \frac{1}{{5108{\text{x}}_{1}^{2} }} - 1 \le 0$$	
	$$q_{3} ({\text{x}}) = 1 - \frac{{140.45{\text{x}}_{1} }}{{{\text{x}}_{2}^{2} {\text{x}}_{3} }} \le 0$$	
	$$q_{4} ({\text{x}}) = \frac{{{\text{x}}_{2} + {\text{x}}_{1} }}{1.5} - 1 \le 0$$	
$\min f(x) = \left( {2\sqrt 2 x_{1} + x_{2} } \right) \times l$	$g_{1} (x) = \frac{{\sqrt 2 x_{1} + x_{2} }}{{\left( {\sqrt 2 x_{1}^{2} + 2x_{1} x_{2} } \right)}}P - \sigma \le 0$	$0 \le x_{i} \le 1,i = 1,2$
	$$g_{2} (x) = \frac{{x_{2} }}{{\sqrt 2 x_{1}^{2} + 2x_{1} x_{2} }}P - \sigma \le 0$$	
	$$g_{3} (x) = \frac{1}{{\sqrt 2 x_{2} + x_{1} }}P - \sigma \le 0$$	

The first engineering optimization problem we chose was: Tension/compression spring design problem (TCSD)^65^, TCSD is a continuously constrained problem such that the volume V of the coil spring is minimized under constant tension/compression load. The second engineering optimization problem we selected is Constrained truss optimization problem^66^. Three-bar truss is a common structural form in engineering, which is widely used in bridges, buildings, mechanical equipment and other fields. The optimization problem of structure design of three-bar truss is to get the best structure layout under certain constraints by adjusting the parameters such as the size, shape and connection mode of the bar. The running results of 12 optimization algorithms in TCSD and Three-bar truss engineering problems are shown in Tables 8 and 9

**Table 8:** The running results of 12 optimization algorithms in TCSD engineering problems.

	IBFO-A	BFO	OOA	GA	GWO	PSO
Best	0.012667247	0.013558966	0.012668072	NAN	0.01266895	0.012666835
Worst	0.014586288	0.029029047	0.016834462	NAN	0.012729057	0.016726958
Average	0.013056487	0.021083917	0.013893121	NAN	0.012705313	0.013185834
Std	4.60e−04	3.59e−03	1.10e−03	NAN	1.95e−05	9.54e−04

	BWO	DBO	COA	GO	AWPSO	ChaoticBFO
Best	0.01273713	0.012674165	0.01267023	0.012669091	0.012666912	0.013769665
Worst	0.016191356	0.017773158	0.01351603	0.017044529	0.014294677	0.023413664
Average	0.013232493	0.013493124	0.012866426	0.013174405	0.012949943	0.013940312
Std	7.39e−04	1.61e−03	1.93e−04	9.22e−04	3.30e−04	4.64e−03

**Table 9:** The running results of 12 optimization algorithms in Three-bar truss engineering problem.

	IBFO-A	BFO	OOA	GA	GWO	PSO
Best	263.8959581	263.9397436	263.9019434	NAN	263.8959079	NAN
Worst	264.0877488	264.8430515	270.2676865	NAN	263.9024999	NAN
Average	263.9072699	264.28099	264.9638793	NAN	263.8977088	NAN
Std	3.01e−02	2.63e−01	1.30e+00	NAN	1.53e−03	NAN

	BWO	DBO	COA	GO	AWPSO	ChaoticBFO
Best	263.9797514	263.8958434	263.895859	263.8958465	263.8958434	263.929491
Worst	264.9257365	263.8969615	263.8975473	263.8966012	263.8963019	265.1599811
Average	264.2141197	263.8959623	263.8962099	263.8959696	263.8958947	264.1313994
Std	1.81e−01	2.00e−04	3.29e−04	1.29e−04	8.55e−05	2.58e−01

Two groups of experiments show that IBFO-A algorithm has improved optimization performance compared with the original BFO algorithm in finding the best objective function. In summary, IBFO-A algorithm has a good optimization ability in practical engineering applications.

### Experimental results and analysis of algorithm convergence

The performance test experiment of the IBFO-D algorithm on network learning is conducted in two steps. In the first step, two types of data were randomly extracted from the alarm benchmark network:

1. Three sets of non-temporal data samples, each containing 1000, 2000, and 3000 randomly selected sample points.
2. Three sets of time series sample data. The time series data contains two time slices, and each set contains 1000, 2000, and 3000 randomly selected sample points.

As the optimization process is a random search, each iteration experiment is independently run 50 times to comprehensively evaluate and analyze the iterative convergence of the fitness values of the IBFO-D algorithm with respect to $B_{0}$ and $B_{ \to }$. This analysis checks the stability of the learning network of the algorithm and whether it falls into its local optimal solution.

We chose to conduct convergence analysis experiments in the alarm network for two reasons:

1. In the field of learning Bayesian network structures, the alarm network is widely recognized as the most popular benchmark.
2. Compared with other network structures, the alarm network is more complex, and its performance on complex networks can better reflect the global search capability and stability of the IBFO-D algorithm.

**Figure 10:**
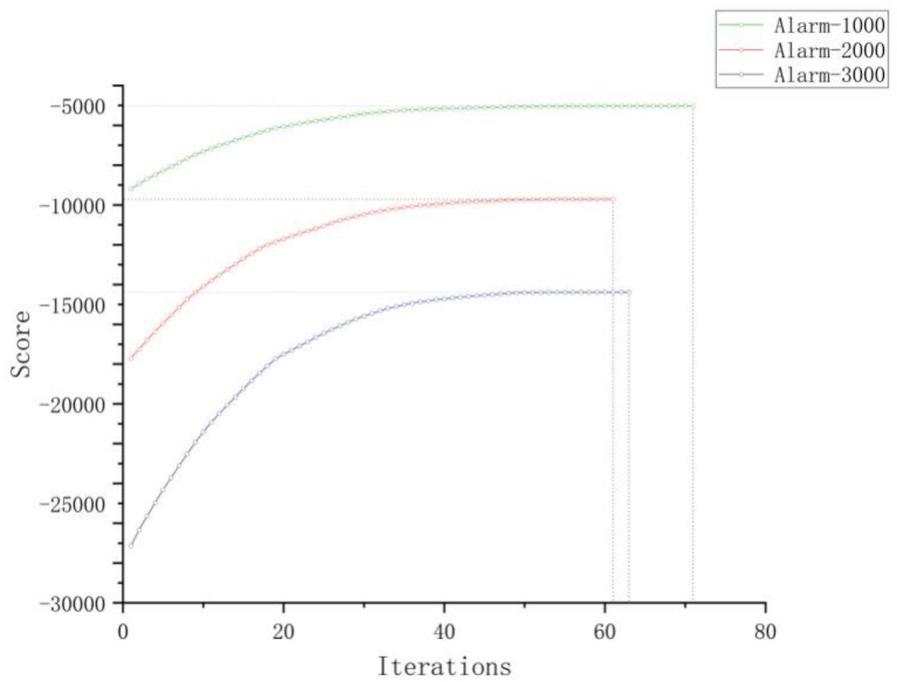
Experimental results of iterative convergence for three sets of non-temporal data samples.

**Figure 11:**
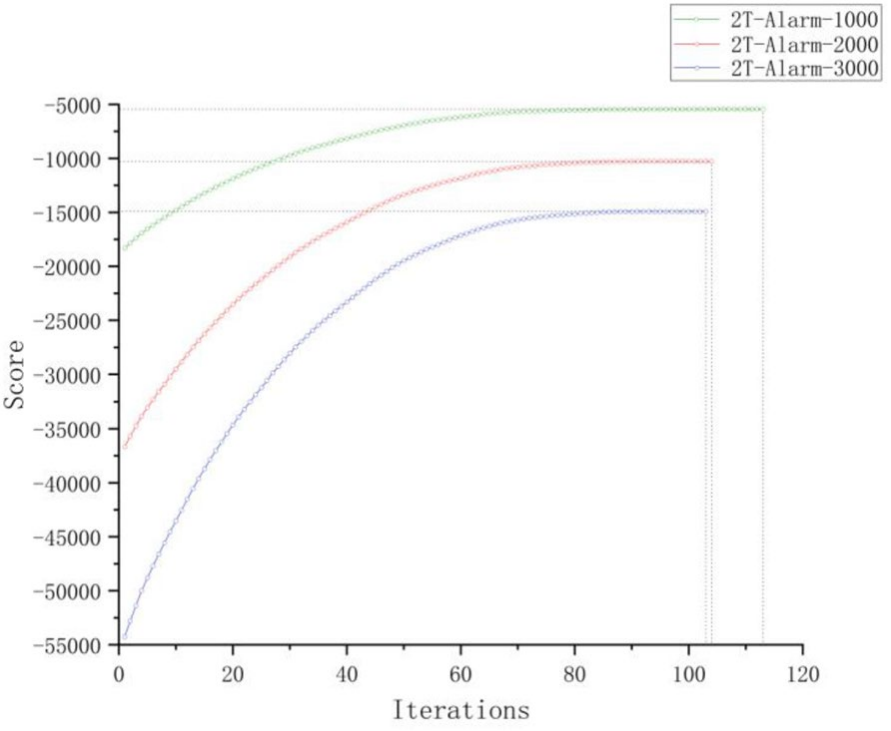
Experimental results of iterative convergence for three sets of 2T-BN temporal data samples.

Figures 10 and 11 illustrate the convergence of fitness scores during iterations, with the X-axis and Y-axis representing the number of iterations and the fitness score, namely the K2 score, respectively. In each generation, the K2 score represents the average outcome of 50 independent runs of the algorithm. An analysis of the experimental findings shows that the algorithm reaches convergence at approximately 110 iterations for the three sets of temporal data, whereas for the other three sets of non-temporal data, convergence is achieved at approximately 70 iterations. This indicates that the IBFO-D algorithm can converge stably within a high fitness value in the temporal and non-temporal data without getting trapped in local optima due to its improved chemotaxis, reproduction, and elimination-dispersal strategies. Furthermore, this algorithm demonstrated a rapid convergence speed and good convergence accuracy.

### Experimental results and analysis of the algorithm performance comparison

The second step involved the use of two dynamic benchmark networks as experimental models. The structural hamming distance (SHD)^67^ was used to compare the IBFO-D algorithm with the DMMHC algorithm^9^ and the GS algorithm^10^ based on temporal information as a comprehensive evaluation metric. This comparison was conducted using three different sets of data samples. Each experiment was independently conducted 50 times to ensure thorough validation of the accuracy and efficiency of these algorithms.

The performance comparison results of the three different algorithms in the small-scale 2T-Asia network and the large-scale 2T-alarm network, across six different data samples, are presented in Tables 10, 11, 12, 13, 14 and 15. In these tables, μ ± σ denotes the average value μ and standard deviation σ of the execution time (seconds) over 50 independent runs for each algorithm.

**Table 10:** Experimental results of the performance comparison of the three algorithms on 2T-Asia-1000.

	DMMHC		GS		IBFO-D	
	SHD	TIME	SHD	TIME	SHD	TIME
Best	2	41.9	8	127.2	2	44.7
Worst	4	63.2	10	191.6	3	61.1
Average	3.5	49.6 ± 11.7	9.3	144.7 ± 15.9	2.2	53.4 ± 6.3

**Table 11:** Experimental results of the performance comparison of the three algorithms on 2T-Asia-2000.

	DMMHC		GS		IBFO-D	
	SHD	TIME	SHD	TIME	SHD	TIME
Best	2	89.8	7	210.7	1	97.6
Worst	3	117.2	10	284.2	2	114.2
Average	2.7	101.3 ± 15.8	7.9	253.3 ± 41.3	1.6	107.4 ± 8.4

**Table 12:** Experimental results of the performance comparison of the three algorithms on 2T-Asia-3000.

	DMMHC		GS		IBFO-D	
	SHD	TIME	SHD	TIME	SHD	TIME
Best	2	182.6	4	392.3	1	198.7
Worst	3	220.3	7	492.6	1	211.8
Average	2.2	196.3 ± 13.3	6.3	441.5 ± 47.1	1	205.2 ± 7.6

**Table 13:** Experimental results of the performance comparison of the three algorithms on 2T-alarm-2000.

	DMMHC		GS		IBFO-D	
	SHD	TIME	SHD	TIME	SHD	TIME
Best	31	523.7	42	712.6	25	645.5
Worst	40	1006.4	61	1326.2	29	752.7
Average	34.7	783.3 ± 256.7	53.3	1026.4 ± 302.4	27.4	697.1 ± 41.4

**Table 14:** Experimental results of the performance comparison of the three algorithms on 2T-alarm-5000.

	DMMHC		GS		IBFO-D	
	SHD	TIME	SHD	TIME	SHD	TIME
Best	24	1546.5	42	2164.1	13	1449.6
Worst	33	2152.8	58	3321.8	20	1813.1
Average	27.6	1798.8 ± 395.3	47.6	2713.5 ± 523.9	18.2	1590.1 ± 184.2

**Table 15:** Experimental results of the performance comparison of three algorithms on 2T-alarm-8000.

	DMMHC		GS		IBFO-D	
	SHD	TIME	SHD	TIME	SHD	TIME
Best	20	2603.6	39	3765.3	9	2414.8
Worst	26	3319.4	45	5143.8	18	2926.7
Average	22.5	3120.5 ± 368.2	42.5	4246.1 ± 663.5	12.3	2753.7 ± 226.1

For the 2T-Asia network, the IBFO-D and DMMHC algorithms have the same optimal SHD when the sample size is 1000. However, the IBFO-D algorithm exhibits better stability and accuracy than the DMMHC algorithm with respect to the worst and average results. This is because the IBFO-D algorithm is based on global-search-based SI optimization, where the error of an individual agent does not affect the optimization outcome of the entire swarm. Furthermore, the reproductive activity in IBFO-D improves the information exchange capability among bacterial individuals, thereby enhancing the overall optimization performance of the bacterial population. (1) From the viewpoint of structural metrics, the differences in algorithm performance become more evident as the sample size increases and network complexity improves. The IBFO-D algorithm exhibits a clear advantage when the sample size is 3000, with a stable SHD of 1. Compared with IBFO-D, the DMMHC algorithm may have lower accuracy, but it generates networks that are relatively close to the true structure. However, the GS algorithm performs the worst, exhibiting the maximum structural variation in all scenarios. (2) The DMMHC algorithm is the fastest, closely followed by the IBFO-D algorithm, while the GS algorithm is the slowest when considering time metrics. These results can be attributed to the fact that global search typically requires more time than greedy local search. Furthermore, in SI algorithms, optimization and complete information exchange tasks are independently executed by individual agents during each iteration, leading to higher time costs. Notably, in the IBFO-D algorithm, significant time savings are achieved by omitting the grouping mechanism when searching for DBN structures in networks such as the small-scale Asia network.

For the 2T-alarm network: (1) When structural metrics are considered, the SHD values for all three algorithms are relatively large in the dataset with a sample size of 2000. This is because a small number of sample cases may not fully reflect the network characteristics in complex networks, leading to challenges in the accurate learning of network structure by the algorithms. However, in all scenarios, the IBFO-D algorithm consistently exhibits smaller structural variations than the other two algorithms, with a worst-case SHD of 29. In the dataset with a sample size of 5000, a notable enhancement in algorithm performance was observed. On average, 91.8 of 110 edges were correctly identified by the IBFO-D algorithm, making it the best-performing algorithm among the three. In the dataset with a sample size of 8000, this advantage becomes even more pronounced. This enhancement is due to the improved chemotaxis and elimination-dispersal approach, which improves the global optimization capability of the IBFO-D algorithm and enables the escape from local optima, thereby facilitating the search for the global optimum structure. (2) Considering time metrics, learning networks in complex node sequences requires more time. Comparative experimental analysis revealed that the IBFO-D and DMMHC algorithms exhibit similar execution efficiencies on large-sample datasets, indicating that the improved chemotactic activity in the IBFO-D algorithm facilitates fast optimization for edge orientation, resulting in optimal time performance. There is one exception, where, in the 2T-alarm-2000 dataset, the DMMHC algorithm outperforms the IBFO-D algorithm in terms of the best runtime. This is due to the lack of a local optima escape mechanism in the DMMHC algorithm, resulting in it being trapped in local optima in complex networks with inadequate sample size. Among the three algorithms, the GS algorithm performs the worst, mainly because of the substantial amount of time spent searching the search space. Based on the aforementioned experimental analysis, it can be concluded that the IBFO-D algorithm is an effective approach for learning DBNs from data, as it can identify network structures with high scores and low structural variations.At the same time, it has high execution efficiency.

## Conclusion

In this study, an IBFO-A was proposed using the logistics-sine chaotic mapping method to initialize the population and improve the chemotactic activity, reproductive activity, and elimination-dispersal activity of the bacteria by combining the OOA algorithm development stage, GA crossover idea, and adaptive method. To solve the problem of complex DBN learning structures due to the introduction of time information, an IBFO-D algorithm is proposed within the framework of the IBFO-A algorithm. In this algorithm, the fitness function and V-structure orientation rule were constructed, and simulation experiments were conducted on a series of reference functions, the 2T-Asia network and the 2T-Alarm network. The experimental results show that the initial population of the IBFOA algorithm using the chaotic mapping method can accelerate the iterative convergence speed, and the improved chemotactic activity and reproductive activity can improve the optimization ability of bacteria. Based on the adaptive elimination-dispersal activity, the algorithm can effectively prevent the local optimal to guide bacteria to find a better solution. The IBFO-D algorithm demonstrates stable convergence at higher fitness values in temporal and non-temporal data, and its performance is better than that of the other two algorithms. Future work will focus on applying the IBFO-D algorithm to learn higher-order dynamic Bayesian networks and time-varying dynamic Bayesian networks to reduce the complexity of their time computation. In addition, the improved BFO method will be combined with other meta-heuristic methods to further improve its ability to search for optimal datasets.

### Supplementary Information

Supplementary Information 1.

Supplementary Information 2.

Supplementary Information 3.

## Data Availability

The datasets generated during and/or analysed during the current study are available from the corresponding author on reasonable request.
