# Supplementary material for: Dynamic Bayesian network structure learning based on an improved bacterial foraging optimization algorithm
Source: Sci Rep. 2024 Apr 9;14:8266. doi: 10.1038/s41598-024-58806-0 (PMC11003998; doi:10.1038/s41598-024-58806-0)
Supplement: Supplementary file 1 — Supplementary Information 1. [file 41598_2024_58806_MOESM1_ESM.doc]

| Networks | Types | Nodes | Edges | Temporal edges | States (min-max) | Parents (min-max) | Neighbors (min-max) |
| --- | --- | --- | --- | --- | --- | --- | --- |
| Asia | $B_{\to}$ | 16 | 21 | 5 | 2-2 | 0-3 | 1-5 |
| Alarm | $B_{\to}$ | 74 | 110 | 18 | 2-4 | 0-4 | 1-7 |
